# Higher estimated net endogenous acid production with lower intake of fruits and vegetables based on a dietary survey is associated with the progression of chronic kidney disease

**DOI:** 10.1186/s12882-019-1591-8

**Published:** 2019-11-21

**Authors:** Koji Toba, Michihiro Hosojima, Hideyuki Kabasawa, Shoji Kuwahara, Toshiko Murayama, Keiko Yamamoto-Kabasawa, Ryohei Kaseda, Eri Wada, Reiko Watanabe, Naohito Tanabe, Yoshiki Suzuki, Ichiei Narita, Akihiko Saito

**Affiliations:** 1 Department of Applied Molecular Medicine, Kidney Research Center, Niigata University Graduate School of Medical and Dental Sciences, Niigata, Japan; 2 Division of Clinical Nephrology and Rheumatology, Kidney Research Center, Niigata University Graduate School of Medical and Dental Sciences, Niigata, Japan; 3 Present Address: Department of Nutrition, Niigata City General Hospital, Niigata, Japan; 4 Department of Clinical Nutrition Science, Kidney Research Center, Niigata University Graduate School of Medical and Dental Sciences, 1-757 Asahimachi-dori, Chuo-ku, Niigata, 951-8510 Japan; 5 Present Address: Laboratory of Clinical Nutrition, Department of Nutrition, School of Human Cultures, The University of Shiga Prefecture, Shiga, Japan; 6 Nutrition Management Unit, Niigata University Medical & Dental Hospital, Niigata, Japan; 7 Present Address: Department of Health and Nutrition, Faculty of Human Life Studies, University of Niigata Prefecture, Niigata, Japan; 8 Department of Health Promotion Medicine, Niigata University Graduate School of Medical and Dental Sciences, Niigata, Japan; 9 Present Address: Laboratory of Metabolic Signal, Institute for Molecular and Cellular Regulation, Gunma University, Gunma, Japan; 10 Department of Health and Nutrition, University of Niigata Prefecture, Niigata, Japan; 11 Health Administration Center, Niigata University, Niigata, Japan

**Keywords:** Dietary acid load, Chronic kidney disease, Fruits and vegetables

## Abstract

**Background:**

Dietary acid load has been suggested to mediate the progression of chronic kidney disease (CKD). However, it is unclear what kinds of foods are actually associated with dietary acid load in patients with CKD. The self-administered diet history questionnaire (DHQ), which semi-quantitatively assesses the dietary habits of Japanese individuals through 150 question items, can estimate average daily intake of various foods and nutrients during the previous month. Using the DHQ, we investigated the association of dietary acid load with CKD progression. We also analyzed the kinds of food that significantly affect dietary acid load.

**Methods:**

Subjects were 96 outpatients with CKD (average estimated glomerular filtration rate [eGFR], 53.0 ± 18.1 ml/min/1.73 m^2^) at Niigata University Hospital, who had completed the DHQ in 2011. We calculated net endogenous acid production (NEAP) from potassium and protein intake evaluated by the DHQ in order to assess dietary acid load. CKD progression was assessed by comparing eGFR between 2008 and 2014.

**Results:**

NEAP was not correlated with protein intake (*r* = 0.088, *p* = 0.398), but was negatively correlated with potassium intake (*r* = − 0.748, *p* < 0.001). Reduction in eGFR from 2008 to 2014 was estimated to be significantly greater in patients with higher NEAP (NEAP > 50.1 mEq/day, *n* = 45) than in those with lower NEAP (NEAP ≤50.1 mEq/day, *n* = 50) by 5.9 (95% confidence interval [95%CI], 0.1 to 11.6) ml/min/1.73 m^2^. According to multiple logistic regression analysis, higher NEAP was significantly associated with lower intake of fruits (odds ratio [OR], 6.454; 95%CI, 2.19 to 19.00), green and yellow vegetables (OR, 5.18; 95%CI, 1.83 to14.66), and other vegetables (OR, 3.87; 95%CI, 1.29 to 11.62).

**Conclusions:**

Elevated NEAP could be a risk factor for CKD progression. Low intake of fruits and vegetables would increase dietary acid load and might affect the progression of renal dysfunction in Japanese CKD patients.

## Background

In chronic kidney disease (CKD), metabolic acidosis is an independent risk factor for the development of end-stage kidney disease [1]. Regardless of CKD stage, administration of sodium bicarbonate slows the decline of kidney function [2, 3]. Physiologically, acid–base balance is affected by meals. Animal products such as meat and cheese are metabolized to sulfate in the body and exert an acidifying effect. Plant products such as fruits and vegetables that are rich in mineral cations and bicarbonate precursors have an alkalizing effect. Excessive continued intake of Western-style diets high in acidifying food is known to induce metabolic acidosis [4]. However, the accumulation of acid may be suppressed by active intake of vegetables and fruits [5]. Dietary acid load, or diet-dependent acid load, can be calculated using information on dietary intake, and net endogenous acid production (NEAP) has been used as its index [6]. To date, several studies have shown the involvement of dietary acid load in the progression of CKD. Studies conducted in Western countries reported low serum bicarbonate concentrations [7] and high rates of decline in estimated glomerular filtration rate (eGFR) [8] among CKD patients with high NEAP. Similarly, in Japan, CKD patients with high NEAP had low serum bicarbonate concentrations, high risk of CKD progression, and low survival rates [9]. However, these studies estimated NEAP from the results of urine collection and no analysis was carried out using information on the foods that were actually ingested. Moreover, a previous study using the Food Frequency Questionnaire (FFQ), which allows estimation of habitual dietary intake in a study population, did not show a correlation between high dietary acid load and the progression of CKD [10]. This may be due to the inaccurate evaluation of dietary acid load by the diet survey. In the present study, we estimated dietary acid load using the self-administered Diet History Questionnaire (DHQ) (Ver. 4.3), which uses food frequency and diet history methodologies. The DHQ yields information on the dietary intake of 150 food and beverage items [11]. The validity of the DHQ, particularly in the Japanese population, has been verified by comparison with the results of the 3-day food record method, 24-h urine collection, serum biomarker-based assays, and the doubly labeled water method [12]. To clarify the kinds of food groups that affect acid load in Japanese patients with CKD, we used the DHQ to estimate NEAP, which is an index of dietary acid load, and to analyze the association between NEAP and changes in kidney function.

## Methods

### Study population

**Fig. 1:**
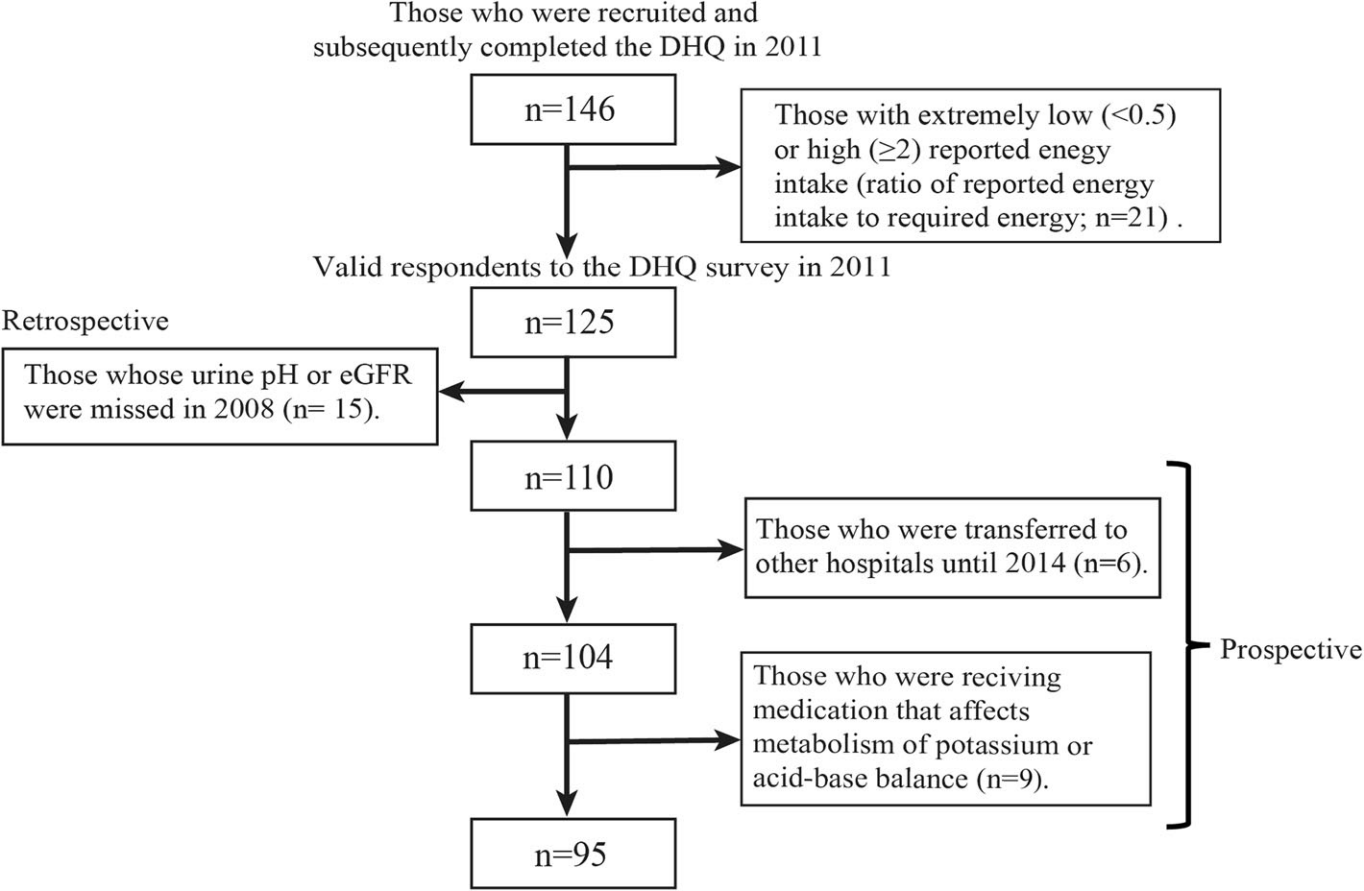
Outline of the protocol for the medical-record-based follow-up study. Results of 95 patients were analyzed. DHQ, self-administered diet history questionnaire; eGFR, estimated glomerular filtration rate

This was a medical-record-based follow-up study of predialysis CKD patients who were treated from 2008 to 2014 at Niigata University Hospital, Japan. The study was approved by the Institutional Ethics Committee of Niigata University (approval number: 1277). The inclusion criteria of this study were outpatients with CKD who were treated at our hospital, who were ≥ 20 years of age, whose eGFR was ≥15 ml/min/1.73 m^2^, and who had provided written informed consent to participate in the study. One hundred and forty-six subjects were recruited and subsequently completed the DHQ in 2011. The study design was a combination of prospective and retrospective elements and was conducted from 2008 to 2014 (Fig. 1). We excluded those subjects with extremely low (< 0.5) or high (≥ 2) reported energy intake (ratio of reported energy intake to required energy; *n* = 21). From the valid respondents to the DHQ survey, those whose urine pH or eGFR was missed in 2008 were retrospectively excluded from the study population (*n* = 15). The remaining 110 subjects were followed until 2014, and those who were transferred to other hospitals until 2014 (*n* = 6) or were receiving medication that affects the metabolism of potassium or acid–base balance (*n* = 9) were excluded from the study [13]. Drugs set as exclusion criteria were calcium polystyrene sulfonate, sodium polystyrene sulfonate, sodium bicarbonate, potassium chloride, and potassium citrate.

### Clinical assessment

Experience of receiving nutrition education between 2008 and 2011 was confirmed from the medical records. Body mass index (BMI) was calculated as weight (kg) divided by height (m) squared. eGFR was calculated as 194 × SCr^-1.094^ × Age^-0.287^ in men and 194 × SCr^-1.094^ × Age^-0.287^ × 0.739 in women [14]. Serum creatinine was calculated by using a kinetic enzymatic method. We defined serum potassium ≥5.5 mEq/L as hyperkalemia. Diabetes mellitus was defined as HbA1c ≥ 6.5% or current use of antidiabetic drugs. Hypertension was defined as systolic blood pressure ≥ 140 mmHg, diastolic blood pressure ≥ 90 mmHg, history of antihypertensive medications, or self-report of hypertension. Urinary protein was assessed using a test strip, and (+) or more was regarded as positive. Urine pH was calculated by using the pH indicator method. We analyzed the change in mean urine pH and eGFR by using laboratory data from September to November 2008, 2011, and 2014.

### Dietary assessment

Dietary habits during the preceding month were assessed in 2011 using the previously validated DHQ [11, 12]. The DHQ is a 22-page semi-quantitative questionnaire that asks about the consumption frequency and portion size of selected foods to estimate the dietary intake of 150 food and beverage items during the preceding month. We asked CKD outpatients who had given their informed consent to complete the DHQ at the hospital or their home. Incomplete entries were confirmed by dieticians, and where there was a blank space or an unrealistic value, the participant was asked to re-answer and described their response. The DHQ consists of 7 sections: (i) general dietary behavior, including preference for seasonings; (ii) usual cooking methods for fish and shellfish, meat, eggs, and vegetables; (iii) consumption frequency and amount of 6 alcoholic beverages; (iv) consumption frequency and semi-quantitative portion size of selected food and non-alcoholic beverage items; (v) type, frequency, and quantity of dietary supplements; (vi) consumption frequency and semi-quantitative portion size of staple foods (rice, other grains, noodles, bread, and other wheat products), soup for noodles and miso (fermented soybean paste) soup, assessed separately for each meal time (breakfast, lunch, dinner, and snacks), with questions on the size of the bowl or cup usually used for rice and miso soup; and (vii) open-ended items for foods consumed more than once weekly, but are not queried in the DHQ [12].

### Estimation of dietary acid load

Several algorithms have been developed to estimate acid load from diet. In this study, we used the method proposed by Frassetto et al., which calculates the dietary protein/potassium ratio in g/mEq [6]: NEAP (mEq/day) = 54.5 × [protein intake (g/day)/potassium intake (mEq/day)) − 10.2.

### Statistical analysis

Numerical variables are expressed as the mean ± standard deviation. Dietary intake of foods and nutrients was assessed in weight per 1000 kcal. Patients were categorized into higher (NEAP > 50.1 mEq/day) or lower (NEAP ≤50.1 mEq/day) NEAP groups based on the mean value of NEAP (50.1 ± 13.1). Differences in patient characteristics between the two groups were examined by Student’s *t*-test for numerical variables and the χ^2^ test for categorical variables: Yates’ correction for continuity was applied for the analysis of 2 × 2 tables. The linear correlation between two variables was assessed by Pearson’s correlation coefficient (*r*).

Repeated-measures analysis of variance was used to assess how NEAP (higher of lower) was associated with longitudinal changes of urine pH and eGFR. When the time × NEAP interaction was not significant (*p* ≥ 0.05), we estimated the overall difference in mean values between the higher and lower NEAP groups as the main effect of NEAP in the model without the interaction term. Furthermore, the changes in eGFR from 2008 to 2011 and 2014 were compared between the higher and lower NEAP groups. The adjusted mean differences were estimated using linear regression models adjusted for sex, BMI (continuous), proteinuria (positive or negative), diabetic status in 2011 (positive or negative), and baseline eGFR in 2008 (continuous). We analyzed how food intake was related to NEAP levels. For that purpose, we made dichotomous variables for the intake (higher or lower) of various food groups based on their mean intake values. Then, crude and adjusted odds ratios (ORs) of the intake of each food group (higher or lower) on NEAP levels (higher or lower) were calculated by unadjusted and multivariable logistic regression; the multivariable model adjusted for intake of other selected food groups. The forward stepwise method (*p*-in < 0.1, *p*-out ≥0.1) was used in order to select food-group variables to be included in the final model from all food-group variables. Statistical tests were performed using SPSS Statistics ver. 17.0 for Windows (SPSS, Inc., Chicago, IL) or IBM SPSS Statistics 21.0 for Windows (IBM Corp., Armonk, NY). A two-tailed *p*-value < 0.05 was considered statistically significant.

## Results

**Fig. 2:**
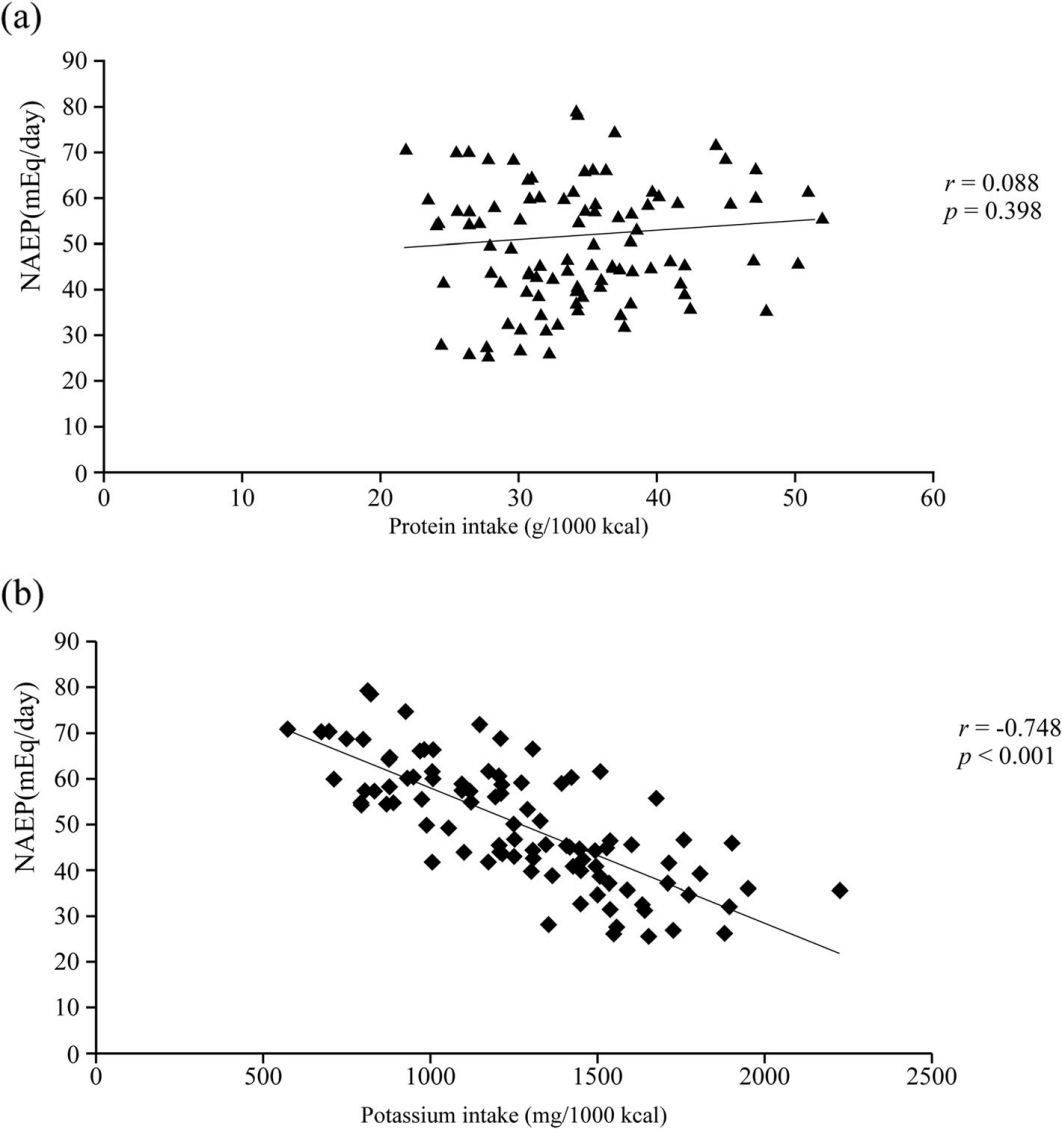
Correlation of NEAP with protein and potassium intake. Scatter plots of NEAP vs. (**a**) protein intake and (**b**) potassium intake. *r*, Pearson’s correlation coefficient. NEAP, net endogenous acid production

**Fig. 3:**
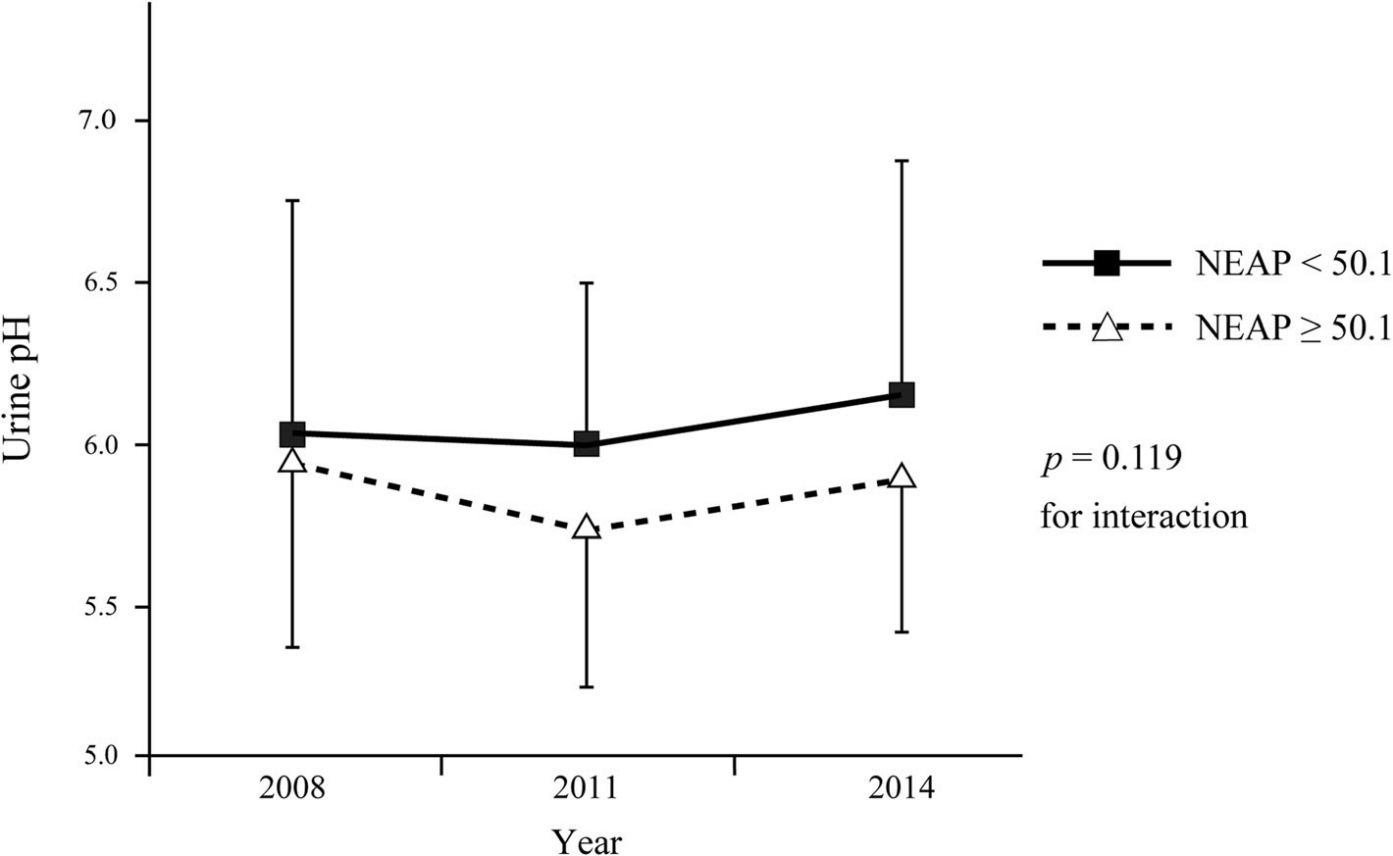
Six-year trends in mean urine pH by NEAP levels. Solid squares and open triangles represent mean values, and vertical lines represent standard deviations. Because the interaction between time and NEAP was not significant, we estimated the overall difference in mean urine pH between the two NEAP groups, which was estimated to be 0.228 (95% confidence interval, 0.044 to 0.412, *p* = 0.016). NEAP, net endogenous acid production

**Fig. 4:**
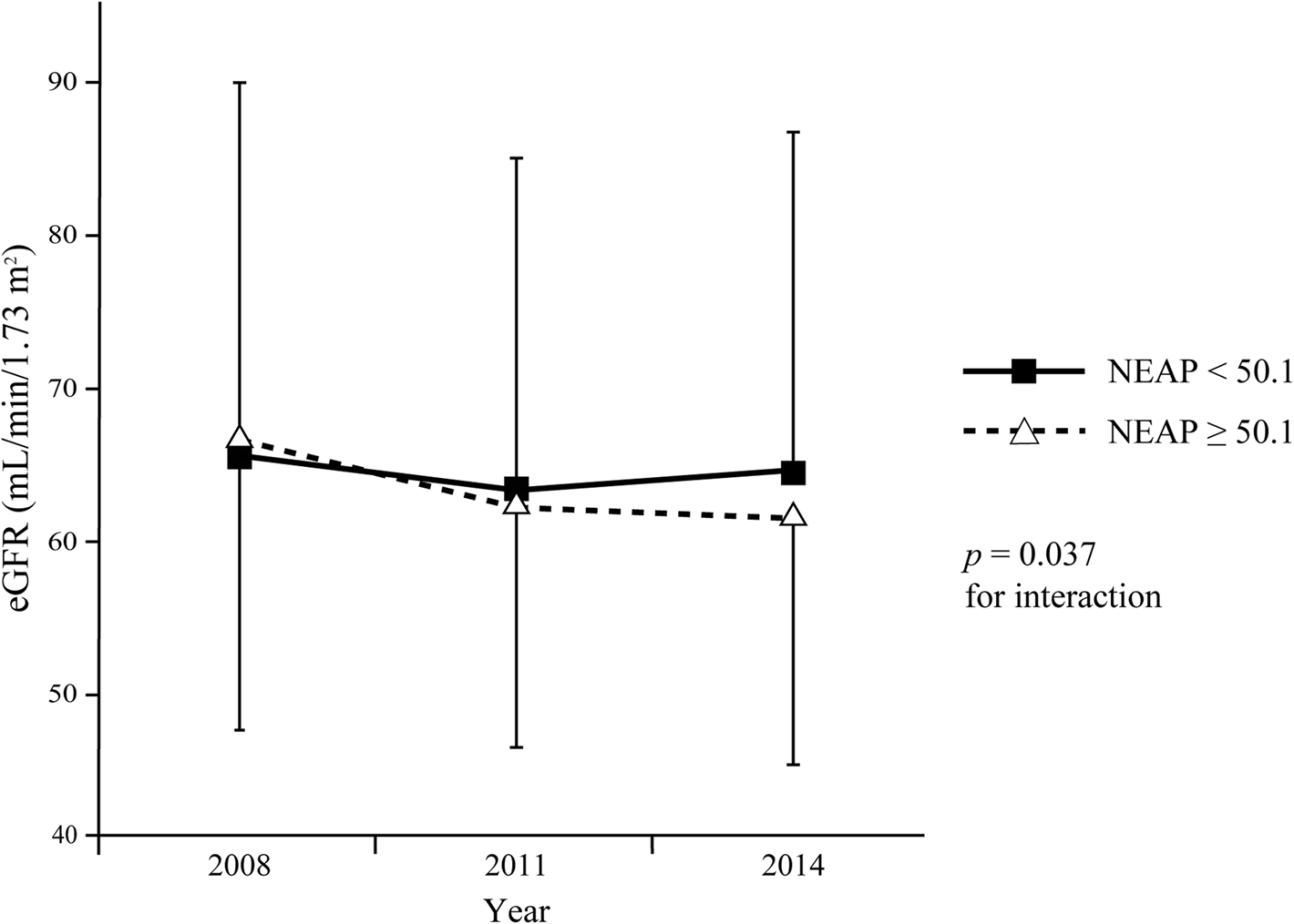
Six-year trends in mean eGFR by NEAP levels. Solid squares and open triangles represent mean values and vertical lines represent standard deviations. eGFR, estimated glomerular filtration rate; NEAP, net endogenous acid production

Mean NEAP was 50.1 ± 13.1 mEq/day. Table 1 shows the characteristics of the study subjects as of 2011. Almost all participants had hypertension. The proportion of male patients and serum levels of sodium and chloride were significantly higher in patients with higher NEAP than in those with lower NEAP. There was no significant difference in age, CKD stage distribution, diabetic status, experience of receiving nutrition education, and hyperkalemia between the two groups. Table 2 shows the energy and nutrient intake of both groups. Energy intake was significantly higher in patients with higher NEAP than in those with lower NEAP. There was no significant difference in protein intake between the two groups, but intake of plant protein and potassium was significantly lower in patients with higher NEAP than in those with lower NEAP. As for other nutrients, the intake of animal fat and cholesterol was significantly higher, and the intake of calcium, magnesium, iron, vitamin A, vitamin B_1_, vitamin B_2_, vitamin C, and total dietary fiber was significantly lower in patients with higher NEAP than in those with lower NEAP. There was no significant difference in sodium intake between the two groups. The correlation of NEAP with protein and potassium intake is shown in Fig. 2. NEAP was not correlated with protein intake (*r* = 0.088, *p* = 0.398; Fig. 2a), but was negatively correlated with potassium intake (*r* = − 0.748, *p* < 0.001, Fig. 2b). We added the analysis for the association between animal or plant protein intake and NEAP levels. This analysis showed that higher NEAP was associated with higher animal protein intake (r = 0.26, *p* = 0.01) and low plant protein intake (r = − 0.31, *p* = 0.002). Figure 3 shows the 6-year trends in mean urine pH. There was no significant interaction between time and NEAP. The estimated overall difference in mean urine pH between the two NEAP groups was 0.228 (95% confidence interval [95%CI], 0.044 to 0.412, *p* = 0.016); that is, mean urine pH was significantly lower in patients with higher NEAP than in those with lower NEAP by 0.228 throughout the study period. Figure 4 shows the 6-year trends in mean eGFR from 2008 to 2014. Mean eGFR was slightly higher in patients with higher NEAP than in those with lower NEAP in 2008 but was lower in 2014. A significant interaction (*p* = 0.037) between time and NEAP was observed; showing that the decline in mean eGFR was significantly greater in patients with higher NEAP than in those with lower NEAP. Table 3 shows the estimated change in mean eGFR from 2008 to 2014 adjusted for sex, BMI, proteinuria, and diagnosis of diabetes in 2011, and baseline eGFR in 2008. The estimated mean change was − 3.5 (95%CI, − 6.1 to − 1.0) ml/min/1.73 m^2^ from 2008 to 2011 and − 2.7 (95%CI, − 6.8 to 1.4) ml/min/1.73 m^2^ from 2008 to 2014 in subjects with lower NEAP, and was − 6.1 (95%CI, − 8.8 to − 3.4) ml/min/1.73 m^2^ and − 8.5 (95%CI, − 12.8 to − 4.2) ml/min/1.73 m^2^, respectively, in patients with higher NEAP. The estimated difference between the two groups in the mean change from 2008 to 2014 was 5.9 (95%CI, 0.1 to 11.6, *p* = 0.045) ml/min/1.73 m^2^; that is, the decline was significantly greater in patients with higher NEAP than in those with lower NEAP by 5.9 ml/min/1.73 m^2^. Table 4 shows the comparison of food intake between patients with higher NEAP and those with lower NEAP. The intake of meat, eggs, and alcoholic beverages was significantly higher and the intake of potatoes, green and yellow vegetables, other vegetables, fruits, mushrooms, and non-alcoholic beverages was significantly lower in patients with higher NEAP than in those with lower NEAP. Table 5 shows the crude and adjusted associations between the intake of each food group and NEAP level. According to crude ORs, a higher intake of nuts and seeds, meats, and eggs and a lower intake of potatoes, fruits, green and yellow vegetables, other vegetables, and mushrooms were associated with higher NEAP (*p* < 0.1). Of these food groups, forward stepwise logistic regression analysis revealed that a lower intake of fruits (adjusted OR, 6.45; 95%CI, 2.19 to 19.00; *p* = 0.001), green or yellow vegetables (adjusted OR, 5.18; 95%CI, 1.83 to 14.66; *p* = 0.002), and other vegetables (adjusted OR, 3.87; 95%CI, 1.29 to 11.6; *p* = 0.016) was significantly associated with higher NEAP, whereas a higher intake of meat tended to be associated with higher NEAP (adjusted OR = 2.64; 95%CI, 0.92 to 7.61; *p* = 0.071).

**Table 1:** Characteristics of study subjects in 2011

		NEAP (mEq/day)		*P*
		< 50.1 (*n* = 50) (25.6–50.1)	≥50.1 (*n* = 45) (50.8–79.2)	
Sex (Male)	n (%)	22 (44.0)	34 (75.6)	0.004
Age	years	66.7 ± 11.6	64.8 ± 11.1	0.427
BMI	kg/m^2^	24.8 ± 4.7	24.8 ± 4.4	0.980
eGFR	ml/min/1.73 m^2^	63.4 ± 17.2	63.0 ± 21.5	0.908
Creatinine	mg/dl	0.9 ± 0.3	1.0 ± 0.4	0.095
CKD stage 1	n (%)	2 (4.0)	4 (8.9)	0.799
CKD stage 2	n (%)	29 (58.0)	24 (53.3)	
CKD stage 3	n (%)	16 (32.0)	14 (31.1)	
CKD stage 4	n (%)	3 (6.0)	3 (6.7)	
Proteinuria	n (%)	21 (42.0)	26 (57.8)	0.183
Diabetes	n (%)	36 (72.0)	25 (55.6)	0.146
Nutrition education	n (%)	6 (12.0)	8 (17.8)	0.615
Hyperkalemia	n (%)	4 (8.0)	1 (2.2)	0.424
Serum uric acid	mg/dl	5.5 ± 1.6	5.8 ± 1.4	0.391
Serum electrolytes				
K	mEq/L	4.2 ± 0.4	4.1 ± 0.3	0.683
Na	mEq/L	139.4 ± 2.6	140.3 ± 1.5	0.037
Cl	mEq/L	102.9 ± 3.0	104.0 ± 2.2	0.048
NEAP	mEq/day	39.5 ± 6.7	61.9 ± 6.8	< 0.001

Data are expressed as the mean ± standard deviation or number (percentage). *BMI* Body mass index, *CKD* Chronic kidney disease, *eGFR* estimated glomerular filtration rate, *Nutrition education* Received nutrition education, *NEAP* Net endogenous acid production

**Table 2:** Comparison of energy and nutrient intake between patients with higher NEAP and those with lower NEAP

		NEAP (mEq/day)		*P*
		≤50.1 (*n* = 50) (25.6–50.1)	> 50.0 (*n* = 45) (50.8–79.2)	
Energy	kcal/day	1558 ± 421	1758 ± 481	0.033
Total protein	% energy	13.8 ± 2.3	13.8 ± 3.0	0.905
Animal protein	% energy	6.4 ± 2.4	7.3 ± 2.7	0.099
Plant protein	% energy	7.4 ± 1.2	6.5 ± 1.5	0.004
Total fat	% energy	22.7 ± 5.9	24.2 ± 6.9	0.253
Animal fat	% energy	8.2 ± 2.9	10.1 ± 3.6	0.006
Plant fat	% energy	12.1 ± 4.1	11.8 ± 4.3	0.722
Cholesterol	mg/1000 kcal	133 ± 53	163 ± 63	0.014
Carbohydrate	% energy	63.5 ± 7.1	61.9 ± 8.6	0.334
Total dietary fiber	g/1000 kcal	9.1 ± 2.4	6.3 ± 1.8	< 0.001
Sodium	mg/1000 kcal	2021 ± 528	1945 ± 451	0.453
Salt equivalent	g/1000 kcal	5.1 ± 1.3	4.9 ± 1.1	0.453
Potassium	mg/1000 kcal	1489 ± 258	1026 ± 241	< 0.001
Calcium	mg/1000 kcal	284 ± 111	234 ± 92	0.018
Magnesium	mg/1000 kcal	150 ± 24	122 ± 29	< 0.001
Phosphorus	mg/1000 kcal	529 ± 94	502 ± 113	0.216
Iron	mg/1000 kcal	4.1 ± 1.0	3.4 ± 0.9	0.002
Zinc	mg/1000 kcal	4.1 ± 0.5	4.1 ± 0.7	0.641
Vitamin A	μg RAE/1000 kcal	313 ± 160	227 ± 169	0.013
Vitamin D	μg/1000 kcal	4.1 ± 2.5	4.3 ± 0	0.653
Vitamin B_1_	mg/1000 kcal	0.45 ± 0	0.39 ± 0	0.001
Vitamin B_2_	mg/1000 kcal	0.71 ± 0.20	0.61 ± 0.20	0.025
Vitamin C	mg/1000 kcal	78 ± 29	38 ± 15	< 0.001

Data are expressed as the mean ± standard deviation. *NEAP* Net endogenous acid production, *RAE* Retinol activity equivalent

**Table 3:** Estimated change in mean eGFR from 2008

Year	Estimated change of mean eGFR (95%CI)		Difference (95%CI)	
	NEAP < 50.1 (*n* = 50)	NEAP ≥50.1 (*n* = 45)		*P*
2011	−3.5 (−6.1, −1.0)	−6.1 (−8.8, − 3.4)	2.5 (−1.0, 6.1)	0.163
2014	−2.7 (−6.8, 1.4)	−8.5 (−12.8, −4.2)	5.9 (0.1, 11.6)	0.045

Unit of eGFR is ml/min/1.73 m^2^; *BMI* Body mass index, *CI* Confidence interval, *eGFR* estimated glomerular filtration rate, *NEAP* Net endogenous acid production

Linear regression model adjusted for sex, BMI, proteinuria, diagnosis of diabetes in 2011, and baseline eGFR in 2008

**Table 4:** Comparison of food intake between patients with higher NEAP and those with lower NEAP

	NEAP (mEq/day)		
	< 50.1 (*n* = 50) (25.6–50.1)	≥50.1 (*n* = 45) (50.8–79.2)	*P*
Cereals	227.9 ± 55.4	248.4 ± 64.6	0.099
Potatoes	25.6 ± 24.2	12.2 ± 6.6	< 0.001
Pulses	38.8 ± 43.1	28.6 ± 23.5	0.150
Green and yellow vegetables	68.9 ± 44.5	33.2 ± 20.7	< 0.001
Other vegetables	90.4 ± 51.9	51.3 ± 27.0	< 0.001
Fruits	120.8 ± 74.2	49.2 ± 34.4	< 0.001
Mushrooms	12.5 ± 10.8	5.3 ± 5.5	< 0.001
Algae	6.5 ± 6.3	5.4 ± 4.5	0.325
Fish and shellfish	39.2 ± 22.6	42.4 ± 26.0	0.529
Meats	21.4 ± 12.9	27.6 ± 14.8	0.031
Eggs	14.2 ± 11.2	19.5 ± 14.0	0.044
Dairy products	69.5 ± 79.0	59.9 ± 53.6	0.485
Pastries	26.4 ± 19.1	23.8 ± 19.1	0.511
Alcoholic beverages	33.2 ± 71.2	80.3 ± 127.2	0.031
Non-alcoholic beverages	516.8 ± 281.1	303.6 ± 227.9	< 0.001

Data are expressed as the mean ± standard deviation (g/1000 kcal). NEAP, net endogenous acid production

**Table 5:** Crude and adjusted odds ratios for higher NEAP according to higher or lower intake of each food group

	Crude			Adjusted			
	OR	(95%CI)	*P*	OR	(95%CI)		*P*
Cereals (higher)	1.23	(0.55–2.75)	0.619				
Potatoes (lower)	3.56	(1.52–8.26)	0.003				
Sugar (lower)	1.04	(0.47–2.34)	0.914				
Pulses (lower)	1.47	(0.65–3.30)	0.353				
Nuts (higher)	2.25	(0.99–5.12)	0.053				
Green and yellow vegetables (lower)	7.94	(3.18–20.00)	< 0.001	5.18	(1.83–14.66)		0.002
Other vegetables (lower)	3.56	(1.52–8.26)	0.003	3.87	(1.29–11.62)		0.016
Fruits (lower)	6.41	(2.62–15.62)	< 0.001	6.45	(2.19–19.00)		0.001
Mushrooms (lower)	2.96	(1.28–6.80)	0.011				
Algae (lower)	1.04	(0.47–2.34)	0.914				
Fish and shellfish (higher)	1.34	(0.60–2.99)	0.476				
Meats (higher)	2.20	(0.99–5.12)	0.053	2.64	(0.92–7.61)		0.071
Eggs (higher)	2.20	(0.99–5.12)	0.053				
Dairy products (lower)	1.04	(0.47–2.34)	0.914				
Fats (higher)	1.16	(0.47–2.34)	0.753				
Oils (lower)	1.24	(0.55–2.78)	0.604				
Pastries (lower)	1.04	(0.47–2.34)	0.914				
Alcoholic beverages (higher)	1.33	(0.59–2.99)	0.489				
Seasoning (higher)	1.13	(0.51–2.54)	0.762				

*CI* Confidence interval, *NEAP* Net endogenous acid production, *OR* Odds ratio

Adjusted ORs were calculated using a multivariable logistic regression model. Variables with *p* < 0.1 for the crude ORs were candidates to be included in the multivariable model. From those candidates, variables for the multivariable model were selected by the forward stepwise method

## Discussion

In this study using the DHQ, we evaluated NEAP, an index of dietary acid load, in Japanese patients with CKD and found that higher NEAP was associated with kidney function decline and was attributable to a lower intake of fruits and vegetables. In the African American Study of Kidney Disease and Hypertension, Scialla et al. estimated NEAP from urine samples collected from African-American patients with stage 3 CKD, and showed low serum bicarbonate concentrations and fast decline in eGFR in patients with high NEAP [7, 8]. Recently, in a cohort study of CKD stages G3 and G4 using data from the National Health and Nutrition Examination Survey, the group with higher dietary acid load assessed by using potential renal acid load, an index of dietary acid load, calculated with the 24-h recall method, was 3 times more at risk for end-stage renal failure than the group with lower dietary acid load [15]. Thus, many reports have suggested that the increase in dietary acid load by a Western diet is associated with the progression of CKD. On the other hand, a report from South Korea demonstrated an association between dietary acid load and CKD in elderly adults [16]. We also reported that higher NEAP was associated with albuminuria, and its association might have a negative relationship with potassium intake in an adult Japanese population (aged ≥40 years) [17]. Also in Japan, Kanda et al. reported that higher NEAP, estimated from collected urine samples, was associated with low serum bicarbonate concentrations, a high risk of CKD progression, and low survival rates among elderly patients with stage 3–5 CKD [9]. The present study was aimed at relatively younger Japanese CKD patients, and used values of NEAP estimated using the DHQ, but the results were similar with these previous studies. Including the results of the present study, similar to reports assessing a Western diet, an increase in dietary acid load due to even an Eastern diet may be associated with the progression of CKD. Each dietary survey has its own limitations. By comparing dietary surveys conducted with CKD patients, Bross et al. showed that the limitations of the 24-h dietary recall method were its dependency on the memory and cooperation of subjects and the skills of questioners, in addition to its lack of information on habitual diet. Furthermore, because of the small number of foods listed in the FFQ, they are often classified into incorrect groups, indicating that the FFQ is not appropriate for the estimation of dietary intake among individuals or in a small population [18]. Tabacchi et al. also reported incorrect classifications associated with the FFQ, and after repeated meta-analyses, they proposed the development of a new FFQ that addresses the need for a valid, reproducible, user-friendly, cost-effective method for accurately assessing nutrient intake in adolescents [19]. Moreover, using the results of the weighed food record method conducted for 28 days as a control, Sasaki et al. showed the low validity of the FFQ due to incorrect classifications and reported that it was difficult to estimate the amount of intake among individuals or in groups with this tool [20]. In the Chronic Renal Insufficiency Cohort Study of patients with CKD and diabetes, Scialla et al. reported that NEAP estimated from collected urine samples was correlated with the progression of CKD, but NEAP estimated using the FFQ was not [10]. These findings suggest that at present, dietary acid load estimations based on information from diet surveys do not give consistent results. Therefore, in this study, we estimated dietary acid load by adopting the DHQ, which was developed by combining items on food frequency and diet history to estimate the dietary intake of 150 different food and beverage items [11]. The validity of the DHQ has been verified by comparison with the outcome of the 3-day food record method, 24-h urine collection, serum biomarkers, and the doubly labeled water method [12], suggesting that the DHQ reflects actual diet more appropriately than the FFQ [11]. As NEAP was estimated from collected urine samples, many previous studies did not analyze the correlation between the progression of CKD and food that was actually ingested. In the clinical setting, it is easier for patients to understand nutritional guidance based on food rather than on nutrients. Therefore, in the present study, we evaluated the strength of the effect of each food group on NEAP. ORs were significantly higher for lower dietary intake of fruits, green and yellow vegetables, and other vegetables. In addition, high dietary intake of meat products tended to increase NEAP, although this was not statistically significant. These results could partly explain the negative correlation between plant protein and NEAP and the positive correlation between animal protein and NEAP. The high consumption of plant protein should be the result of the high consumption of plant-based foods such as beans, vegetables, and fruits. There is more potassium in the main sources of plant protein, such as beans, vegetables, and fruits, than in the main sources of animal protein, such as meat, milk and dairy products, and fish. This tendency has not been observed in studies conducted in Western countries [4, 21] and may be attributable to the Japanese diet (Eastern diet), which does not routinely include consumption of large quantities of meat, milk, and dairy products. Using the Heart-Wise Dietary Habits Questionnaire, Wai et al. conducted a dietary survey of patients with stage 3–4 CKD. Their results revealed that adequate intake of fruits and vegetables and limited alcohol consumption delayed the induction of dialysis and improved patient survival [22]. Also, Wesson et al. showed that consumption of fruits and vegetables is expected to further decrease acid load, thus improving acidosis and reducing acid accumulation in patients with stage 2 CKD compared with those with stage 1 CKD, and they recommended active intake of fruits and vegetables by patients with stage 1 and 2 CKD [5, 23]. The findings of this study suggest that even in patients with stage 2 CKD and relatively stable kidney function, intake of fruits and vegetables, which does not increase NEAP, can effectively suppress the progression of kidney impairment. However, further studies are needed to clarify the amount of fruits and vegetables required for patients with different CKD stages. Conversely, adherence to dietary instructions to reduce NEAP may increase potassium intake, as seen in the lower NEAP group in our study. As the stage of CKD advances, hyperkalemia may develop because of a reduction in urinary potassium excretion and an increase in the frequency of administration of renin-angiotensin system antagonists. Such cases require careful monitoring of serum potassium concentrations. This study has some limitations. Because we did not measure the concentration of serum bicarbonate in this study, we were unable to verify whether metabolic acidosis mediated the association between dietary acid load and CKD progression. However, because urine pH is significantly lower in individuals with higher NEAP, we speculate that the early-stage CKD patients with higher NEAP in this study might have had potential acid retention. Recently, Goraya et al. reported that acid retention is inversely related to GFR, even in early stage CKD patients without metabolic acidosis [24]. Further studies are needed to distinguish the acid-base effects and non-acid-base effects, such as improvements in the intestinal flora, increased magnesium intake, and relatively decreased phosphorus intake, due to fruit and vegetable intake on the renal function of CKD patients. Because this was an observational study, cause-and-effect relationships were not elucidated. Also, some data were obtained retrospectively, so there could be some variability in measurements or definitions, and many variables such as smoking and socioeconomic status were not available. Moreover, this was a single-institution study with a small number of subjects, and the exclusion of 51 subjects may have resulted in study bias. Therefore, we plan to perform a prospective multicenter study that involves a higher number of subjects and includes the measurement of serum bicarbonate concentrations.

## Conclusion

In this study, we evaluated dietary acid load among Japanese patients with CKD using data from the DHQ. In addition to showing a correlation between the progression of CKD and dietary acid load, our findings suggest that higher NEAP could be a risk factor for CKD progression and lower intake of fruits and vegetables is a factor that affects acid load. A prospective study is needed to investigate whether the reduction of dietary acid load suppresses the progression of CKD.

## Data Availability

The datasets used and/or analyzed during the current study are available from the corresponding author on reasonable request. The homepage of the Department of Social and Preventive Epidemiology at The University of Tokyo can be accessed at http://www.nutrepi.m.u-tokyo.ac.jp/english/dhq/dhq.html.

## Abbreviations

BMI: Body mass index
CI: Confidence interval
CKD: Chronic kidney disease
DHQ: Diet History Questionnaire
eGFR: estimated glomerular filtration rate
FFQ: Food Frequency Questionnaire
NEAP: Net endogenous acid production
OR: Odds ratio
RAE: Retinol activity equivalent
